# Outcomes of Rotator Cuff Repair: Open vs. Arthroscopic Approaches in Patients with Diabetes or Hyperlipidemia

**DOI:** 10.26502/josm.511500201

**Published:** 2025-05-15

**Authors:** Kevin Babakhan Vartanian, Kevin Ghookas, Tony Eskandar, Niayesh Najafi, Devendra K. Agrawal

**Affiliations:** Department of Translational Research, College of Osteopathic Medicine of the Pacific, Western University of Health Sciences, Pomona, California 91766, USA

**Keywords:** Arthroscopy, Diabetes mellitus, Hyperglycemia, Hyperlipidemia, Post-operative complications, Rotator cuff injury, Rotator cuff repair

## Abstract

Rotator cuff injuries are a common cause of shoulder dysfunction, with diabetes mellitus and hyperlipidemia contributing to increased tendon vulnerability and impaired healing. In this article, a critical evaluation is presented on the comparative outcomes of open versus arthroscopic rotator cuff repair (RCR) in patients with these metabolic conditions. Findings suggest that arthroscopic RCR compared to open RCR offers advantages such as reduced tissue disruption, shorter recovery times, and lower infection rates, making it a preferred choice for metabolically compromised patients. However, open RCR provides superior visualization and structural support, as well as better function, benefiting patients with extensive tendon damage. In diabetics there was higher retear and infection rates than non-diabetics, particularly with open RCR, while those with hyperlipidemia exhibited impaired tendon healing and increased postoperative complications, with mixed effects from statin therapy. Despite these findings, existing research lacks large-scale, controlled studies directly comparing surgical outcomes in these high-risk populations. Given the chronic inflammatory and metabolic impairments associated with diabetes mellitus and hyperlipidemia, surgical decisions should be tailored to patient-specific factors, including lipid and glycemic control, tendon integrity, and tear severity. Arthroscopy appears to be the preferable option due to minimizing surgical trauma, lower retear rates and faster return-to-work times; meanwhile, open repair remains valuable in cases requiring extensive intervention.

## 1. Introduction

The rotator cuff is a complex and vital anatomical structure within the shoulder, composed of four muscles that play a key role in maintaining stability throughout its full range of motion [1–3]. It is instrumental in facilitating essential movements such as abduction, external rotation, and internal rotation [4,5]. Due to its broad range of motion and inherent biomechanical demands, the rotator cuff is highly susceptible to injury, particularly when the shoulder is subjected to repeated stress in an overhead position [6,7]. This vulnerability makes rotator cuff injuries (RCIs) one of the most common causes of shoulder pain and dysfunction. These injuries can vary in etiology, arising from slow, age-related degenerative processes, or acute trauma during strenuous physical activity such as sports [7–9]. Systemic causes are also a major contributor to RCI, with various metabolic syndromes and genetic susceptibilities increasing risk of tendon injury through chronic tendinopathies [10,11]. Large-scale population studies have shown that individuals with conditions such as diabetes mellitus (DM) or hyperlipidemia (HLD) are at an increased risk for developing rotator cuff injuries, demonstrating a link between chronic metabolic syndrome and a dysregulation of tendon homeostasis [12–21].

In the context of diabetes mellitus (DM), there is growing evidence suggesting that the disease contributes to a higher incidence of rotator cuff injuries, likely due to its detrimental effects on the muscles and tendons at the cellular level [1,16]. Research using rat models of type II diabetes mellitus has revealed that the condition leads to significant structural change to the extracellular matrix, primarily manifesting as abnormal crosslinking of Type I collagen as a result of chronic hyperglycemic conditions and build-up of advanced glycation end products (AGEs) [22–26]. In comparison to control rats, DM modeled rats demonstrated decreased collagen deposition alongside increased collagen degradation, which can confer increased risk of injury due to inherent structural weakness of the tendons [27]. These cellular disruptions could explain the heightened vulnerability of individuals with DM to developing rotator cuff pathologies, highlighting the importance of early detection and management of shoulder health in these populations.

Many published articles have also shown an association between HLD and tendon weakness [28–33]. Biomechanical and tensile testing on tendons in a mouse model have demonstrated a link between HLD and weaker tendons in elderly mice, and a propensity to spontaneous tendon rupture in young mice [34]. In a swine model of hyperlipidemia, we reported severe inflammation, fatty infiltration, and amplified mitochondrial activity that could be contributing to pathophysiological alterations in rotator cuff tendon matrix components leading to decreased biomechanical properties [28–33]. HLD can cause pathologic lipid infiltration and macrophage activity at the tendons, decreasing structural integrity and increasing degradation respectively. A shift from type I to type III collagen within tendons is also observed in chronically elevated lipid states, which can be associated with decreased elastic modulus and greater proclivity to injury [31–34]. These findings, in addition to studies highlighting a higher incidence of rotator cuff tears in patient populations with HLD, suggest susceptibility to tendon injury in HLD and other associated lipid disorders [28,35]. In experimental models, non-surgical strategies have been investigated in modulating the immune response and rotator cuff tendon regeneration following injury [36–39].

**Figure 1:**
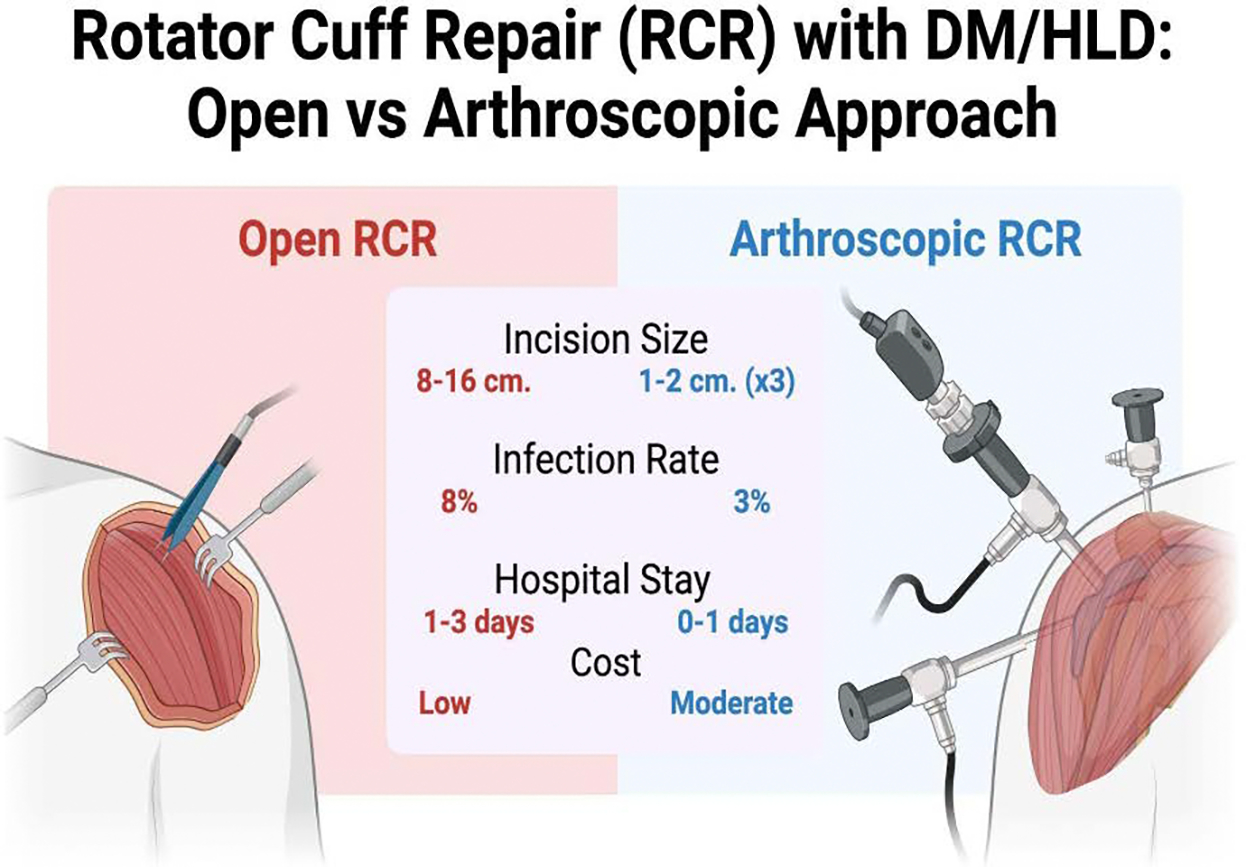
Summary of Open vs. Arthroscopic approaches comparing incision size, infection rate, hospital stay and cost. The illustration depicts the two surgical approaches as well as the common surgical tools used.

The goal of this structured review is to assess the comparative effectiveness of open versus arthroscopic rotator cuff repair surgeries in patients with underlying conditions such as diabetes mellitus (DM) or hyperlipidemia (HLD). Open rotator cuff repair surgery, when compared to the minimally invasive arthroscopic technique, is characterized by a significantly larger incision, typically ranging from 8 to 16 centimeters in length. This surgical approach is often preferred for repairing larger tears, as it allows the surgeon greater visibility and access to the injured area. In contrast, arthroscopic surgery involves the use of several small incisions (usually 1 to 2 centimeters in diameter) through which a small camera (arthroscope) and specialized instruments are inserted to repair the damaged tissue [40]. This minimally invasive technique, although more difficult and time-consuming, theoretically offers several advantages, including reduced tissue disruption, better functional outcome, shorter hospital stay, and reduced risk of complications requiring follow-up surgery [41–43]. A summary of these advantages is depicted in Figure 1. The use of the arthroscope provides real-time images that guide the surgeon’s movements, improving precision and minimizing damage to surrounding structures. Despite the clear differences in the surgical approaches, some studies have indicated that there are no significant long-term differences between the outcomes of open and arthroscopic rotator cuff repairs on a generalized patient basis [44,45]. Specifically, when examining long-term factors such as pain levels, the prevalence of retears, and the risk of infections, the results are often not statistically significant between the two methods [46–48]. Patient satisfaction and subjective scores post open or arthroscopic RCR were also not found to be significantly different in some studies [49].

Taking HLD and DM into account, further investigation is warranted into the benefits of arthroscopic versus open rotator cuff surgery. Given the chronic inflammatory states usually present in these patient populations, one must consider the impaired capacity for healing and increased risk of postoperative complications [50,51]. Therefore, it is critical to determine the optimal surgical approach, and analyze the utility of these two surgical methods in metabolically compromised individuals [52–54]. Analysis of the risks and benefits of these surgical procedures can provide insight into ways to improve recovery and long-term functional outcomes in these high-risk groups following RCI.

## 2. Methods

To evaluate the outcomes of open versus arthroscopic rotator cuff repair surgery in patients with DM and HLD, a comprehensive literature review was conducted. The process included defining specific search terms and setting inclusion and exclusion criteria to focus on studies comparing the two surgical approaches in this patient population. Key articles were selected based on their investigation of surgical outcomes, including pain relief, functional recovery, retear rates, and complication rates in individuals with comorbid DM and HLD. Particular attention was given to studies addressing the influence of these comorbidities on surgical healing, postoperative complications (e.g., infection, delayed wound healing), and the impact on long-term shoulder function. Articles that did not provide comparative analysis or did not address outcomes specifically related to diabetes and hyperlipidemia were excluded. The search was performed using PubMed and Cochrane. Keywords and phrases utilized included “open vs arthroscopic rotator cuff repair,” “rotator cuff repair diabetes,” “hyperlipidemia and rotator cuff surgery,” “arthroscopy surgery hyperlipidemia,” “diabetes shoulder surgery outcomes,” and “arthroscopic surgery complications diabetes.” The review was limited to English-language studies published between 2000 and 2024.

## 3. Open vs. Arthroscopic Approach to Rotator Cuff Surgery

Rotator cuff integrity plays a crucial role in the proper function of the rotator cuff, which is essential for maintaining shoulder strength and mobility. The two most common surgical approaches for treating rotator cuff injuries are arthroscopic surgery and open surgery. The advent of arthroscopic techniques in rotator cuff repair has significantly transformed surgical outcomes and patient experiences. These techniques allow for same-day surgeries, reduced recovery times, and a notably lower number of surgical anchors used compared to traditional open surgery [55]. In addition to these benefits, arthroscopic procedures are associated with shorter return-to-work times and retear rates, which also contributes to a reduction in the overall wait time between the date of injury and recovery [56,57]. This efficiency in surgical intervention can be particularly beneficial in expediting patient care and improving access to treatment [58].

Interestingly, data comparing the two surgical methods revealed no significant difference in retear rates, although these findings did not account for variables such as patient comorbidities or the severity of rotator cuff tears. Some small sample size papers do however suggest arthroscopic repair has statistically significant lower retear rates in comparison to open surgery [57,59–62]. This limitation highlights the need for more nuanced studies to better understand the factors influencing surgical outcomes. Postoperative outcomes, as measured by the American Shoulder and Elbow Surgeons (ASES) scores, demonstrated that patients undergoing arthroscopic surgery reported significantly higher scores six months after the procedure [63,64]. The ASES score is a standardized metric ranging from 0 to 100 that evaluates pain levels and the ability to perform daily activities. While these scores primarily rely on subjective patient reports, they provide valuable insight into patient satisfaction and functional recovery. These outcomes underline the growing preference for minimally invasive techniques in the field of rotator cuff repair [65,66].

Open surgery is not inferior however, and some studies indicate better outcomes for open surgeries as opposed to arthroscopic. A 2020 prospective cohort study comparing arthroscopic and open surgeries of the rotator cuff showed no statistical significance in postoperative pain or range of motion between the two groups, but showed increased function only in the open surgery cohort [67]. In surgical cases where acromioplasty was needed, open surgical methods reflected more favorable outcomes in comparison to arthroscopy [67]. Furthermore, research in the UK has shown open surgery to be less costly, with the added benefit of shorter average surgery time [53,54]. Overall, the research shows that the decision between arthroscopy versus the classical open surgery is more tailored to the individual patient and specifics of the injury, rather than a statistical difference in outcomes, reflecting the popularity of both approaches.

## 4. Diabetes Considerations

**Figure 2:**
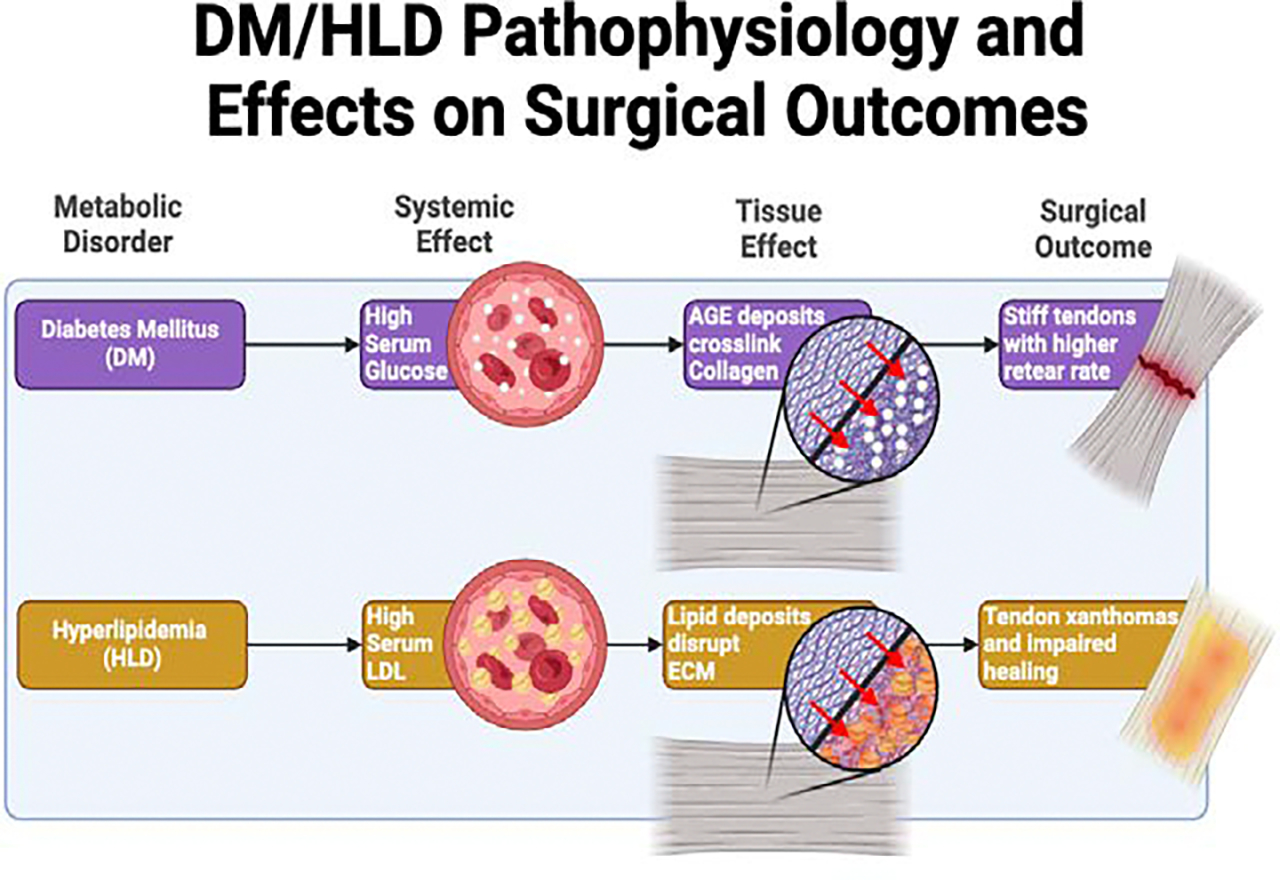
Impact of diabetes mellitus and hyperlipidemia on tissue healing. Figure depicts pathophysiology of tendon structure due to comorbidities and impact on postoperative complications.

Postoperative rotator cuff repair complications including retears, failure, and infection have been found to be higher in patients with DM [68]. Furthermore, poor glycemic control in DM patients undergoing rotator cuff repair is shown to impact healing rates [69]. Hemoglobin A1C (HbA1C) is a measure of glycosylated hemoglobin in circulation and is an established marker of glycemic control. High HbA1c levels such as DM are correlated with higher levels of microangiopathy which impacts blood vessels and the ability for tissues to receive nutrients and oxygen which are paramount in postoperative healing. Higher HbA1c measured from 1 month preoperatively to 3–6 months postoperatively was correlated with a statistically significant higher retear rate [70,71]. Furthermore, AGE deposits crosslink collagen which stiffens the tendon and results in higher incidence of tears and retears, as shown in Figure 2 [1,22,24,26,72,73].

Arthroscopic surgery, although more complex in its execution, allows for a more minimally invasive approach leading to less damaged tissue and shorter postoperative time spent in the hospital in comparison to open surgery [42,74]. This decreased healing demand places less stress on the already compromised healing capabilities of diabetes patients and therefore is theoretically more applicable to DM patients versus unaffected patients. In one study showing the comparative difference between patients with and without diabetes undergoing arthroscopic repair, there was a statistically significant decrease in functional outcomes scores and ASES scores for diabetic patients at 6 months post-op [75]. This highlights the impact of diabetes on recovery even with the minimally invasive nature of the procedure.

Forward elevation and external rotation between 6 months and year post operatively reflected a wider separation between diabetic and non-diabetic patients compared to earlier time periods. This points to the presence of long-term healing insufficiencies in DM, rather than just a slower rate of recovery, which further grounds the statistics of retear rates [76]. Open surgeries with DM patients showed an increase in failure rate from 3% to 7%, but the sample sizes were too small to make statistical comparisons [68]. Furthermore, this cohort was under insulin control which is shown to decrease the retear rates and therefore does not allow for a direct comparison with the previously mentioned studies [77,78]. Open surgery also has a higher incidence of infection rates, which is why most DM studies with rotator cuffs focus on arthroscopy, as infections are more common in diabetic patients [43,52]. Although there is no literature with direct comparisons and minimal heterogeneity amongst techniques, patient populations, and level of control of the disease, the literature favors arthroscopic approaches as the benefits of open surgery do not outweigh the increased demand on healing, and the higher infection rates.

## 5. Hyperlipidemia Considerations

**Figure 3:**
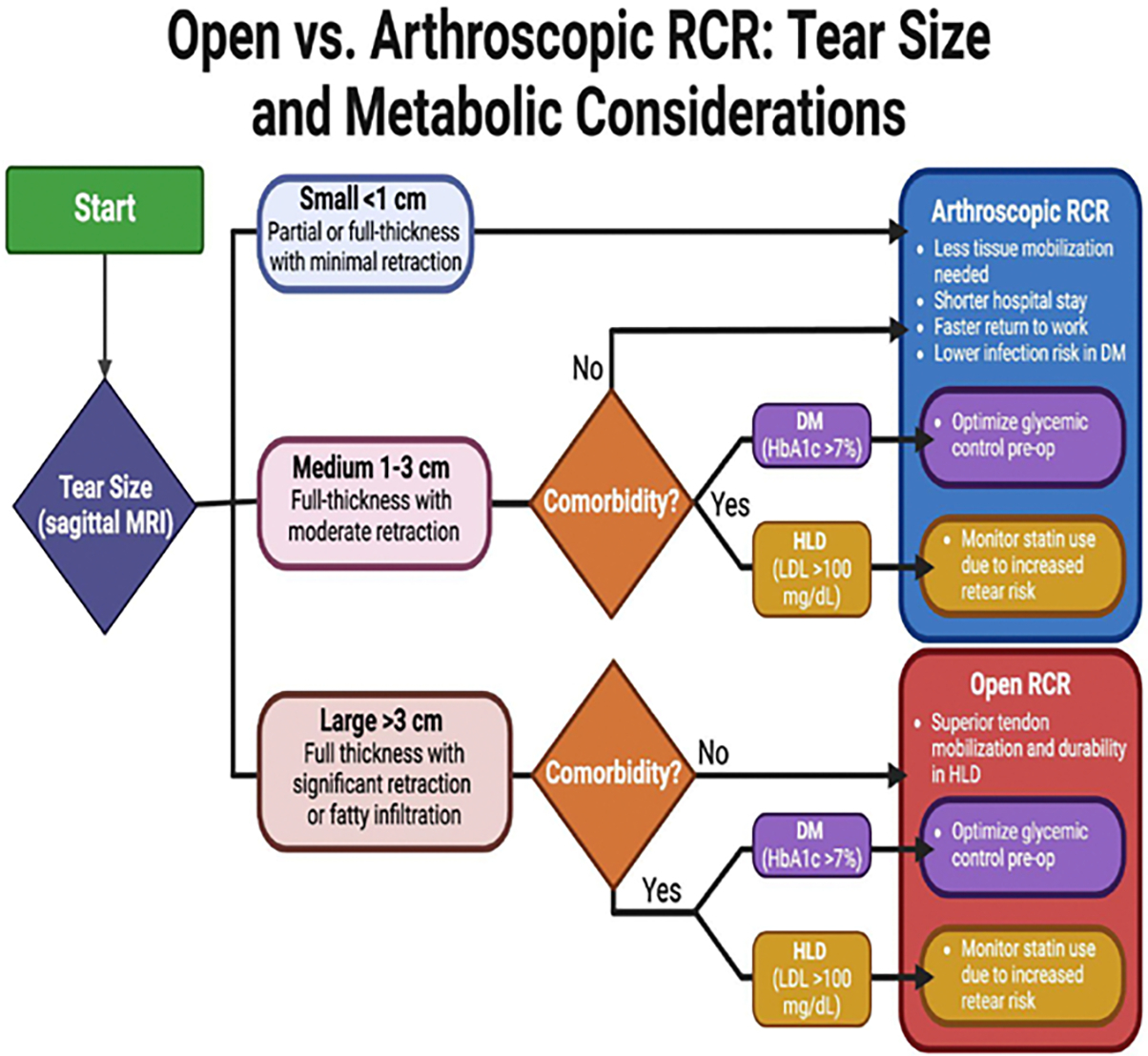
Summarized decision-making tree for surgical approach taking into account tear size, if a comorbidity is present and finally important factors to control such as statins and glycemic control.

Hyperlipidemia (HLD) is another comorbidity that impacts postoperative success in rotator cuff surgeries. Hyperlipidemia is the elevation of total cholesterol, LDL, and triglycerides which occurs through a polygenic and lifestyle etiology [31–33,79]. Research has shown a correlation not only between worsened postoperative success of rotator cuff surgeries in patients with HLD, but also increased rates of initial rotator cuff injury [35]. Higher serum lipid levels cause lipid depositions called xanthomas in various tissues including but not limited to tendons, eyelids, and other musculoskeletal regions. These xanthomas disturb the extracellular matrix and impact multiple aspects of homeostasis ranging from vasculature to protein synthesis and can in turn increase the likelihood of injury [79–81]. This relationship is depicted in Figure 2. Other studies have shown no correlation between serum cholesterol levels and incidence of joint injuries, and debate is still ongoing on the connection between the two [82]. However, the correlation between healing rates and elevated serum cholesterol has been repeatedly demonstrated [83–85]. The role of statins is complicated in terms of its influence on the tendons and muscle and therefore will be discussed later. Due to the impact on healing, arthroscopic surgery inherently is beneficial as the minimally invasive nature results in overall lower tissue damage and therefore less stress on the body to heal. However, this does not mean that arthroscopic surgery circumvents these healing problems. HLD in arthroscopic surgery has been shown to result in statistically significant levels of postoperative complications in comparison to healthy patients [86,87]. Furthermore, a retrospective review determined that HLD patients on statin medications had significantly higher rate of retears in arthroscopic RCI surgery compared to healthy individuals suggesting the mechanism in which statins reduce serum cholesterol does not completely reverse the diminished healing capacity in HLD [78]. Statins are still very effective and controlling them is important for post operative success, which is highlighted in Figure 3 [88].

Despite the benefit of a decreased healing demand gained from a minimally invasive approach, open surgery also has been shown to have a multitude of benefits. In larger or more chronic tears, open surgery via larger incision can mobilize retracted tendons and lead to better functional outcomes [80]. Although no research has been done to directly compare these two approaches in patients with HLD, no definitive answer does not suggest that they are identical but rather a more patient specific consideration is needed. These decisions will depend on the size of tear, severity of the comorbidity, and expertise of the surgeon due to the higher incidence of retears for patients with hyperlipidemia [56,89–91]. Both surgical outcomes have their benefits and drawbacks, although there is an increased popularity towards arthroscopic surgery. This is due to the shorter hospital stay times and lower retear rates, in addition to less healing stress on the already compromised rotator cuff [92,93]. Despite the lack of concrete evidence, the data points to arthroscopic surgery providing an overall better probability of success and patient satisfaction. The ultimate decision requires many considerations tailored to the patient and the surgeon’s preference, as well as the exact context of the injury, location, size, and previous history of injury [94–96]. A summary of the decision-making steps is shown in Figure 3, with considerations for the size of the tear and comorbidities factored in. Although these are not hard-set rules, they provide a reference point for decision making in cases of poor glycemic control or HLD.

## 6. Future Directions

While substantial progress has been made in understanding the comparative effectiveness of open versus arthroscopic rotator cuff repair (RCR), notable gaps persist, especially concerning patients with comorbidities like diabetes mellitus (DM) and hyperlipidemia (HLD). Current research tends to focus on generalized patient populations without accounting for the nuanced effects these conditions have on postoperative healing, retear rates, and overall patient outcomes.

Future studies should prioritize large-scale, multicenter randomized controlled trials (RCTs) specifically targeting the impact of DM and HLD on surgical outcomes. These trials should stratify patients based on glycemic and lipid control levels to better elucidate how disease severity influences recovery. Furthermore, longitudinal studies with extended follow-up periods (beyond 12 months) are necessary to determine the sustainability of both surgical methods in patients with these metabolic disorders.

## 7. Drawbacks

Despite the expanding body of literature comparing open and arthroscopic rotator cuff repair (RCR), several significant limitations persist. A primary drawback is the lack of disease-specific data, as most studies do not account for comorbidities like diabetes mellitus (DM) and hyperlipidemia (HLD), which can considerably influence healing and surgical outcomes. This oversight limits the generalizability of findings to high-risk patient populations who often experience different recovery trajectories [56,96,97]. Another challenge lies in the variability of surgical techniques and surgeon experience across studies. Differences in surgical approach, rehabilitation protocols, and postoperative care can introduce considerable heterogeneity, making direct comparisons between studies difficult and potentially skewing outcome interpretations. Some studies included in this review circumvented this by using identical methods with one surgeon, but this is at the cost of sample size as these papers never exceeded more than 30 patients. Furthermore, the feasibility of performing randomized control trials while controlling for all these factors may prove to be exceedingly difficult.

Additionally, many studies suffer from short-term follow-up periods, often focusing on outcomes within six months of surgery. This short-term perspective may overlook crucial long-term factors such as retear rates, chronic shoulder dysfunction, and patient satisfaction over extended periods. Compounding this issue is the subjectivity of functional outcome measures like the American Shoulder and Elbow Surgeons (ASES) score. These patient-reported metrics can be influenced by individual perceptions and are further heightened in populations with comorbidities.

Another drawback is the underrepresentation of diverse patient populations in current research [98,99]. Most studies are conducted in high-income countries with limited mention of patient population metrics, neglecting how socioeconomic status, cultural differences, and disparities in access to rehabilitation services can affect outcomes [62,100,101]. Finally, there is a lack of comprehensive long term cost-effectiveness and patient satisfaction data [102]. Although minimally invasive techniques such as arthroscopy are presumed to offer faster recovery and higher patient satisfaction, the long-term economic implications, especially in cases requiring reoperations or extended rehabilitation, are inadequately explored. Addressing these limitations through more robust, standardized, and inclusive research will be essential for advancing personalized surgical care in patients with DM and HLD.

## 8. Conclusions

This review highlights the complexities surrounding the choice between open and arthroscopic rotator cuff repair (RCR) in patients with comorbidities such as diabetes mellitus (DM) and hyperlipidemia (HLD). Both surgical methods have distinct advantages and limitations, with outcomes often depending on patient-specific factors, including the severity of comorbidities, tendon quality, and tear complexity [103]. Arthroscopic repair offers benefits like reduced tissue disruption and quicker recovery times making it particularly advantageous for patients seeking minimally invasive options. However, the technical demands of arthroscopy and increased risk of complications in certain high-risk populations, such as those with poorly controlled DM or HLD, underscore the need for careful patient selection.

Conversely, open RCR provides superior visualization and the ability to manage larger or more complex tears, which can be crucial for patients with chronic degenerative changes commonly seen in HLD or longstanding DM. Despite being more invasive, and therefore placing further stress on the impacted healing capabilities, open surgery may offer more durable repairs in cases of significant tendon degeneration or poor tissue quality [104]. It has also been shown to be more cost effective along with shorter average surgery time. Importantly, several studies indicate that long-term outcomes—such as retear rates, pain relief, and functional recovery—do not significantly differ between the two approaches for the general population, though data specific to patients with DM and HLD remains limited.

The information in this article underscores the necessity for individualized planning that accounts for comorbidities, metabolic control, and patient preferences. The decision-making process should involve a thorough discussion between the patient and surgical team, weighing the risks and benefits of each approach [104,105].

Ultimately, the findings highlight a critical gap in literature regarding the comparative effectiveness of these surgical techniques in metabolically compromised populations. Future large-scale, multicenter randomized controlled trials (RCTs) with extended follow-up are essential to better define optimal surgical strategies for patients with DM and HLD. By addressing these gaps, healthcare providers can improve postoperative outcomes and provide more personalized, evidence-based care for this high-risk patient population.

## 7. Key Points

- Metabolic conditions like diabetes mellitus (DM) and hyperlipidemia (HLD) significantly impair tendon health, healing, and increase the risk of rotator cuff injuries and surgical complications.
- Arthroscopic rotator cuff repair (RCR) is generally favored in patients with DM or HLD due to its minimally invasive nature, resulting in shorter hospital stays, lower infection risks, and faster recovery.
- Open RCR offers better visualization and is often more effective for large, complex, or chronic tears, particularly where extensive tendon retraction is present.
- DM patients experience higher retear and infection rates, particularly with open surgery. Poor glycemic control (e.g., elevated HbA1c) is strongly associated with poor healing outcomes.
- HLD is linked to structural tendon changes (e.g., xanthomas, Type I → III collagen shift) and higher postoperative complication rates, with mixed effects observed from statin therapy.
- Existing literature lacks large, disease-specific randomized controlled trials (RCTs) that directly compare open vs. arthroscopic RCR outcomes in DM/HLD populations, creating a significant evidence gap.
- Many studies are limited by small sample sizes, short-term follow-ups, and lack of disease stratification, which hampers their applicability to real-world high-risk patients.
